# Use of a visceral protective layer prevents fistula development in open abdomen therapy: results from the European Hernia Society Open Abdomen Registry

**DOI:** 10.1093/bjs/znad163

**Published:** 2023-06-14

**Authors:** Sebastian Schaaf, Robert Schwab, Aliona Wöhler, Filip Muysoms, Johan F Lock, Karl Sörelius, Rene Fortelny, Tobias Keck, Frederik Berrevoet, Gregor A Stavrou, Martin von Websky, Dario Tartaglia, Dirk Bulian, Arnulf Willms

**Affiliations:** Department of General, Visceral and Thoracic Surgery, German Armed Forces Central Hospital Koblenz, Koblenz, Germany; Department of General, Visceral and Thoracic Surgery, German Armed Forces Central Hospital Koblenz, Koblenz, Germany; Department of General, Visceral and Thoracic Surgery, German Armed Forces Central Hospital Koblenz, Koblenz, Germany; Department of Surgery, Maria Middelares Hospital, Ghent, Belgium; Department of General-, Visceral-, Transplant-, Vascular- and Paediatric Surgery, University Hospital of Würzburg, Würzburg, Germany; Department of Vascular Surgery, Rigshospitalet, University of Copenhagen, Copenhagen, Denmark; Faculty of Health and Medical Sciences, University of Copenhagen, Copenhagen, Denmark; Department of General, Visceral and Oncological Surgery, Vienna, Austria; Medical Faculty, Sigmund Freud University of Vienna, Vienna, Austria; Department of Surgery, University Hospital Schleswig-Holstein (UKSH), Lübeck, Germany; Department of General and Hepatopancreatobiliary Surgery and Liver Transplantation, Ghent University Hospital, Ghent, Belgium; Department of General, Visceral and Thoracic Surgery, Surgical Oncology, Klinikum Saarbrücken, Saarbrücken, Germany; Department of General, Visceral, Thoracic and Vascular Surgery, University Hospital Bonn, Bonn, Germany; General, Emergency and Trauma Surgery Unit, Pisa University Hospital, Pisa, Italy; Department of Abdominal, Tumor, Transplant and Vascular Surgery, Cologne-Merheim Medical Centre, Witten/Herdecke University, Cologne, Germany; Department of General, Visceral and Vascular Surgery, German Armed Forces Hospital Hamburg, Hamburg, Germany

## Introduction

Open abdomen therapy has become a commonly used strategy in the treatment of serious abdominal conditions. A potential major complication is the development of enteroatmospheric fistula, as it has been linked to increased mortality and morbidity^1,2^. Vacuum-assisted wound closure and mesh-mediated fascial traction has been demonstrated to be a safe technique when applied correctly^3,4^. To reduce the risk of fistula development, the use of an inert film (visceral protective layer, VPL) between the vulnerable intestines and dressing material/mesh might be beneficial^4,5^. This study aimed to define the effect of VPL application on the incidence of enteroatmospheric fistula during open abdomen therapy based on a large registry data set.

## Methods

### European Hernia Society (EHS) Open Abdomen Registry

In May 2015, the European Hernia Society (EHS) Open Abdomen Registry was implemented as a module of the European Registry of Abdominal Wall Hernias to allow collection and analysis of data from patients with an open abdomen^6,7^. As of 2021, the registry has been relocated to https://www.ehs-openabdomen.com, and has been updated; a detailed description is available in the *supplementary material*. All patients documented in the EHS Open Abdomen Registry between its establishment on 1 May 2015 and 4 December 2022 were enrolled in this study. Informed consent was obtained. The Ethics Committee of the State Chambers of Medicine in Rhineland-Palatinate approved the study (837.534.13 (9219-F), which was registered in the International Clinical Trials Registry platform through the German Registry for Clinical Trials (DRKS00031181).

### Study design and analysis

A propensity score matched case–control study (1 : 1 treatment : controls, caliper width 0.2) was conducted to reduce the risk of confounding. The intervention group (VPL) included all patients who received open abdomen therapy including a VPL, whereas the control group (no VPL) comprised the remainder. The selection of variables for matching was based on both clinical relevance and descriptive statistics, and included malignant disease, arterial hypertension, renal disease, haemodialysis, pulmonary disease, evidence of risk factors for a complicated course, open abdomen therapy as initial surgical procedure, number of previous operations, duration of open abdomen therapy, and attainment of fascial closure.

Continuous data are presented as mean(s.d.). Data were analysed using descriptive statistical methods, with differences between groups examined by use of the χ^2^ test or Mann–Whitney *U* test. *P* < 0.050 was considered significant. Logistic regression analysis with enteroatmospheric fistula as the dependent variable was undertaken, whereas potential influencing factors were defined as independent variables. The data were processed and analysed in Microsoft^®^ Excel version 365 (Microsoft, Redmond, WA, USA), XLSTAT^™^ (Addinsoft, Paris, France^Tumor^), and SPSS^®^ version 20 (IBM, Armonk, NY, USA).

## Results

The registry contained data from 1009 patients; characteristics of the overall and matched populations are shown in *Table S1*, and surgical management and outcomes in *Tables 1* and *S2–S4*. Peritonitis was the commonest indication for inclusion in the registry (36.6 per cent). Open abdomen therapy was based on a structured approach in 369 patients (37.3 per cent), and at the surgeon’s individual discretion in 620 (62.7 per cent).

**Table 1:** Indications for and outcomes of open abdomen therapy

	Unmatched population				Propensity score-matched population		
	All (*n* = 1009)	Visceral protective layer (*n* = 561)	No visceral protective layer (*n* = 448)	P*	Visceral protective layer (*n* = 198)	No visceral protective layer (*n* = 198)	P*
Open abdomen therapy as initial surgical procedure	446 (44.2)	272 (48.5)	174 (38.8)	0.001	0 (0)	0 (0)	–
No. of previous operations, mean(s.d.)	1.6(0.9)	1.5(0.8)	1.7(1.0)	0.012†	1.5(0.7)	1.7(1.0)	0.101†
Emergency index operation	275 (27.3)	153 (27.3)	122 (27.2)	0.310	80 (40.8)	83 (46.9)	0.305
**Indication for open abdomen**							
Peritonitis	369 (36.6)	209 (37.3)	160 (35.7)	0.606	88 (44.9)	71 (35.9)	0.067
Burst abdomen/abdominal compartment syndrome	298 (29.5)	158 (28.2)	140 (31.3)	0.287	54 (27.6)	81 (40.9)	0.007
Trauma	89 (8.8)	62 (11.1)	27 (6.0)	0.012	12 (6.1)	14 (7.1)	0.711
Other	253 (25.1)	132 (23.5)	121 (27.0)	0.214	42 (21.4)	32 (16.2)	0.178
Fascial separation at commencement of therapy (cm), mean(s.d.)	13.7(6.9)	14.4(7.1)	11.9(6.3)	0.002†	13.6(6.3)	11.5(5.9)	0.068†
Negative-pressure wound therapy	687 (68.1)	508 (90.6)	179 (40.0)	0.001	173 (88.3)	100 (50.5)	0.002
Negative-pressure wound therapy intensity (mmHg), mean(s.d.)	79.1(35.4)	90.2(31.9)	51.9(28.4)	0.004†	92.6(31.1)	53.0(27.7)	0.001†
Dynamic closure techniques	693 (68.7)	382 (68.1)	311 (69.4)	0.653	140 (71.4)	167 (84.3)	0.001
**No. of open abdomen dressing changes**							
0–5	605 (68.1)	400 (76.0)	205 (56.5)	0.001	133 (70.4)	107 (59.4)	0.013
6–10	21 (2.4)	3 (0.5)	18 (5.0)	0.001	0 (0)	9 (5.0)	0.001
11–15	218 (24.5)	102 (19.4)	116 (32.0)	0. 002	50 (26.5)	54 (30.0)	0.692
> 15	45 (5.1)	21 (4.0)	24 (6.6)	0.223	6 (3.2)	10 (5.6)	0.306
Duration of open abdomen therapy (days), mean(s.d.)	20.8(37.0)	18.3(36.2)	24.1(37.8)	0.021†	20.8(42.7)	23.9(31.7)	0.407†
In-hospital death	282 (27.9)	168 (29.9)	114 (25.4)	0.111	46 (23.5)	53 (26.8)	0.446
Duration of hospital stay (days), mean(s.d.)	57.6(66.0)	54.5(61.2)	61.5(71.5)	0.118†	62.3(62.6)	58.0(39.8)	0.432†
Fascial closure	561 (55.6)	369 (65.8)	192 (42.9)	<0.001	149 (76.0)	108 (54.5)	0.001
Enteroatmospheric fistula development	71 (7.0)	23 (4.1)	48 (10.7)	<0.001	12 (6.1)	32 (16.3)	<0.001
Enteroatmospheric fistula (failed fascial closure)	39 (8.7)	14 (7.3)	25 (9.8)	0.013	8 (16.3)	17 (35.6)	0.065
Time to fistula development (days), mean(s.d.)	23.2(20.3)	29.6(26.7)	20.5(16.5)	0.131†	29.1(27.9)	20.2(16.8)	0.281†

Values are *n* (%) unless otherwise indicated. See *Tables S2–S4* for additional data. *χ^2^ test, except †Mann–Whitney *U* test.

In both populations, there were no significant differences in sex, age, or BMI. Around 70–79 per cent of patients reported co-morbidities, and the no-VPL group had a slightly higher proportion before and after propensity score matching. This resulted in a less pronounced difference in the prevalence of arterial hypertension between the VPL and no-VPL groups (38.8 *versus* 30.8 per cent; *P* = 0.102).

Approximately 20 per cent of the overall population had malignant disease, with the VPL group showing higher proportions before and after matching. However, the severity of co-morbidity scores was not significantly different after matching. In the total cohort, 32.5 per cent of patients reported risk factors for complications while undergoing open abdomen therapy, such as concurrent use of immunosuppressants and/or anticoagulants. The initial fascial dehiscence, measured as the maximal horizontal width between the fascial edges, was significantly greater in the VPL than in the no-VPL group before matching (mean(s.d.)14.4(7.1) *versus* 11.9(6.3) cm; *P* = 0.002). Even after matching, there was still a difference between the two groups, although it was not statistically significant.

A VPL was used in 561 patients (55.6 per cent). Propensity score matching revealed two equally powered groups of 198. Negative-pressure wound therapy was used widely; however, it was applied more often in the VPL group (matched groups: 88.3 *versus* 50.5 per cent; *P* = 0.002). Dynamic closure techniques were applied in 68.7 per cent of patients, with no relevant difference between unmatched VPL and no-VPL groups; however, after matching a difference was observed (71.4 *versus* 84.3 per cent; *P* = 0.001). The duration of open abdomen therapy was 20.8(37.0) days overall, with no significant difference between groups. Similar results were seen for the number of dressing changes. Establishing open abdomen therapy was the initial surgical procedure in 44.2 per cent of patients, with a higher proportion in the VPL group (48.5 per cent *versus* 38.8 per cent in no-VPL group; *P* = 0.001). Interestingly, propensity matching led to exclusion of all such patients.

The overall mortality rate was recorded as 27.9 per cent, with no meaningful variation with VPL use. The same held true for duration of hospital stay and time spent on ventilator in critical care. Fascial closure was reported in 55.6 per cent overall, with the VPL group showing higher rates both before and after propensity matching (matched groups: 76.0 *versus* 54.5 per cent; *P* = 0.001).

**Fig. 1:**
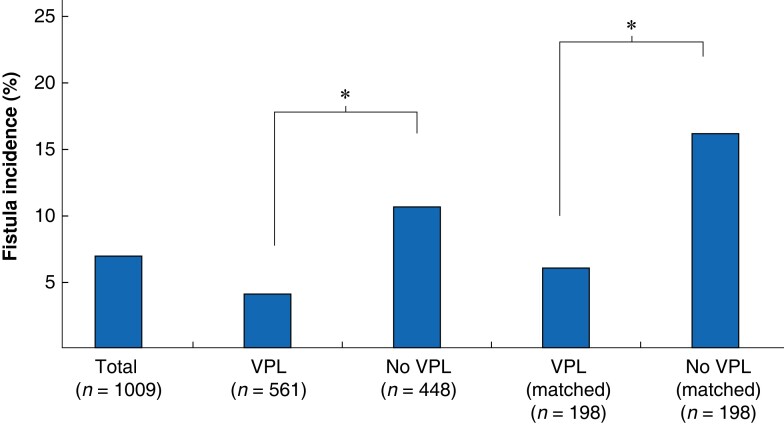
Enteroatmospheric fistula rates with and without application of a visceral protective layer before and after propensity score matching VPL, visceral protective layer. *P < 0.001 (χ^2^ test).

Seventy-one enteroatmospheric fistulas (7.0 per cent) were reported in the registry. The VPL group had significantly lower enteroatmospheric fistula rates than the no-VPL group (4.1 *versus* 10.7 per cent in unmatched groups, *P* < 0.001; 6.1 *versus* 16.3 per cent in matched groups, *P* < 0.001) (*Fig. 1*) The interval from commencement of open abdomen therapy to development of enteroatmospheric fistula was 23.2(20.3) days. Thirty-nine enteroatmospheric fistulas (55 per cent) occurred in patients with failed fascial closure; however, reduced rates of fistula were noted in the VPL group even if fascial closure was not achieved (7.3 *versus* 9.8 per cent in unmatched groups, *P* = 0.013; 16.3 *versus* 35.6 per cent after matching, *P* = 0.065).

Regression analysis revealed that VPL had a significant impact on enteroatmospheric fistula formation with an OR of 0.36 (95 per cent c.i. 0.21 to 0.60; *P* < 0.001; *R*²=0.05) for the overall cohort, and 0.34 (0.17 to 0.68; *P* = 0.002; *R*²=0.04) after propensity score matching.

## Discussion

The enteroatmospheric fistula rate reported here is in line with rates of 3–19 per cent reported in other studies^3,8–10^. An enteroatmospheric fistula is a severe potential complication of open abdomen therapy associated with increased morbidity, mortality, and impaired quality of life^1^.

The effect of negative-pressure wound therapy in open abdomen treatment and the risk of fistula development is debated; however, a shorter duration of open abdomen therapy combined with dynamic closure techniques has been shown to be both safe and effective^3,11,12^.

There are few available data on the effect of VPL on fistula incidence. This issue was investigated previously in a smaller study of open abdomen therapy after peritonitis^5^. In that study, a remarkable 10-fold risk reduction in enteroatmospheric fistula development was shown when a VPL was used. Moreover, another recent analysis from the EHS Open Abdomen Registry^12^ found the combination of negative-pressure wound therapy, VPL, and dynamic closure techniques to be beneficial in terms of achieving fascial closure and thus reducing complications. This was also addressed in the 2018 EHS clinical guidelines^4^ on the management of the abdominal wall in the context of open and burst abdomen. A pooled incidence of enteroatmospheric fistula of 4.3 per cent was reported for dynamic closure techniques *versus* 11.9 per cent for static closure techniques, and the use of dynamic closure techniques in uncomplicated open abdomen therapy (Björck grades I and II) was therefore recommended^4^. When dynamic closure techniques are used, in particular mesh-mediated traction, it is imperative to consider that direct contact between the mesh material and oedematous or granulating intestinal serosa without a VPL should be avoided because of the significant risk of complications^5,12^.

In conclusion, use of a VPL should be considered in open abdomen therapy. If incorporated into a standardized treatment approach and in conjunction with negative-pressure wound therapy and dynamic closure techniques, the time taken for open abdomen therapy can be reduced, and the likelihood of complications, particularly enteroatmospheric fistula, can therefore be reduced.

## Supplementary Material

znad163_Supplementary_Data

## Data Availability

The data will be available upon request to the corresponding author.
