# Supplementary material for: Use of a visceral protective layer prevents fistula development in open abdomen therapy: results from the European Hernia Society Open Abdomen Registry
Source: Br J Surg. 2023 Jun 14;110(12):1607–10. doi: 10.1093/bjs/znad163 (PMC10638526; doi:10.1093/bjs/znad163)
Supplement: znad163_Supplementary_Data [file znad163_supplementary_data.docx]

**The Use of a Visceral Protective Layer Prevents Fistula Development in Open Abdomen Therapy: Results from the European Hernia Society Open Abdomen Registry**

Sebastian Schaaf^1^, Robert Schwab^1^, Aliona Wöhler^1^, Filip Muysoms^2^, Johan F. Lock^3^, Karl Sörelius^4,5^, Rene Fortelny^6,7^, Tobias Keck^8^, Frederik Berrevoet^9^, Gregor A. Stavrou^10^, Martin von Websky^11^, Dario Tartaglia^12^, Dirk Bulian^13^, Arnulf Willms^14^

^1^ Department of General, Visceral and Thoracic Surgery, German Armed Forces Central Hospital Koblenz, Rübenacher Str. 170, 56072 Koblenz, Germany

^2^ AZ Maria Middelares, Buitenring Sint-Denijs 30, 9000 Gent, Belgium

^3^ Department of General-, Visceral-, Transplant-, Vascular- and Pediatric Surgery, University Hospital of Würzburg, Würzburg, Germany

^4^ Department of Vascular Surgery, Rigshospitalet, University of Copenhagen, Blegdamsvej 9, 2100, Copenhagen, Denmark.

^5^ Faculty of Health and Medical Sciences, University of Copenhagen, Blegdamsvej 3B, 2200, Copenhagen, Denmark

^6^ Department of General, Visceral and Oncological Surgery, Wilhelminenspital, 1160, Vienna, Austria.

^7^ Medical Faculty, Sigmund Freud University of Vienna, 1020, Vienna, Austria.

^8^ Department of Surgery, University Hospital Schleswig-Holstein (UKSH), Campus Lübeck, 23538 Lübeck, Germany.

^9^ Department of General and HPB Surgery and Liver Transplantation, Ghent University Hospital, Corneel Heymanslaan 10, 9000, Ghent, Belgium.

^10^ Department of General, Visceral and Thoracic Surgery, Surgical Oncology, Klinikum Saarbrücken, Saarbrücken, Germany.

^11^ Department of General, Visceral, Thoracic and Vascular Surgery, University Hospital Bonn, Bonn, Germany.

^12^ General, Emergency and Trauma Surgery Unit, Pisa University Hospital, 56124 Pisa, Italy.

^13^ Department of Abdominal, Tumor, Transplant and Vascular Surgery, Cologne-Merheim Medical Center, Witten/Herdecke University, Ostmerheimer Str. 200, 51109, Cologne, Germany.

^14^ Department of General, Visceral and Vascular Surgery, German Armed Forces Hospital Hamburg, Lesserstraße 180, 22049 Hamburg, Germany

**Corresponding author:**

Sebastian Schaaf, Department of General, Visceral and Thoracic Surgery, German Armed Forces Central Hospital Koblenz, Rübenacher Str. 170, 56072 Koblenz, Germany

Tel: +49 261 22810, email: sebastianschaaf1@bundeswehr.org

ORCID: 0000-0002-1920-7090

**Supplementary Materials - Index**

| **Supplementary Methods** |  |
| --- | --- |
| The EHS Open Abdomen Registry | *page 2* |
| **Supplementary Results** |  |
| Comprehensive discussion of the study’s results | *page 3* |
| **Supplementary Figures and Tables** |  |
| Tables S1-S4 | *page 6* |
| **References** | *page 10* |

**Supplementary Methods**

## The EHS Open Abdomen Registry

In May 2015, the European Hernia Society (EHS) open abdomen registry was implemented as a module of the European Registry of Abdominal Wall Hernias (EuraHS) by a working group of surgeons from the Department of General, Visceral, Transplantation, Vascular and Paediatric Surgery at the University Hospital of Würzburg and the Department of General, Visceral and Thoracic Surgery at the Federal Armed Forces Central Hospital in Koblenz. The EuraHS platform was developed by the Department of Artificial Intelligence and Applied Informatics of the Institute for Mathematics and Computer Science at the University of Würzburg in Germany. [1] The registry was created to allow data to be collected and analysed from patients with an open abdomen from multiple centres in a systematic and standardised manner and thus to achieve a higher level of evidence. [2]

As of 2021, the registry has been updated and relocated to www.ehs-openabdomen.com. To make data entry easier and more accurate, the questions and variables have been simplified based on previous analyses. The updated registry of open abdomen management includes nine categories of data collection, including information on hospitals and patient-specific factors, comorbidities, risk factors for complications (immunosuppression and/or anticoagulants), indication and management of the open abdomen, fascial closure, discharge and follow-up.

Three key questions concerning the management of the open abdomen are addressed: which material has direct contact to the intestines, are dynamic fascial closure techniques/fascial traction applied, and is negative-pressure wound therapy used. Additional information can be entered depending on the indication for open abdomen therapy, such as the injury severity score, Mannheim Peritonitis Index, and APACHE II. The intraoperative findings are scored according to the Björck classification. With the registry update, the amended Björck classification has been applied. [3]

**Supplementary Results**

## Comprehensive discussion of the study results

### Summary of main study results

This study provides data from the EHS Open Abdomen Registry regarding the overall population and the propensity score matched collective. Out of 1009 patients, 561 (55.9%) underwent an open abdomen therapy technique that included a VPL. Negative-pressure wound therapy was employed in 687 (68.1%) cases, with a higher proportion in the VPL group. Dynamic closure techniques with fascial traction was utilised in 693 (68.7%) cases, with no significant differences between the VPL and no VPL groups. Only 369 (37.3%) of the cases followed a standardised open abdomen therapy approach, with the remainder relying on the individual judgement of the surgeon. The number of patients with enteroatmospheric fistula was 71 (7.0%) and the interval from open abdomen therapy commencement to the occurrence of the fistula was 23.2 ± 20.3 days. The regression analysis revealed a significant risk reduction of 66% for enteroatmospheric fistula development if a VPL was used for open abdomen therapy (OR=0.34, CI: 0.17-0.68, p=.002).

### Enteroatmospheric fistula rates

The enteroatmospheric fistula rate presented here is in line with data from other studies. Cristaudo et al. published a recent paper on predicting factors for enteroatmospheric fistula based on a dataset of 300 patients and reported an enteroatmospheric fistula incidence of 8.4%. [4] In other studies, the incidence of enteroatmospheric fistula formation in open abdomen therapy ranged from 5-19%, with particularly high rates in series with open abdomen therapy for peritonitis. [5–9] Interestingly, a recent analysis from the International Registry of Open Abdomen (IROA) based on 1183 cases revealed a low overall enteroatmospheric fistula rate of 2.5%. [10] It is worth noting that the median open abdomen therapy duration of the cases in the IROA was four days, compared to 11 days in our study. In an earlier analysis from the IROA, the enteroatmospheric fistula incidence was 8.9% with a mean (SD) open abdomen duration of 7.9 (18.22) days, which is still less than half the duration of our analysis. [11]

### Negative effects of enteroatmospheric fistulae

The development of enteroatmospheric fistula in the open or granulating laparostomy is not only detrimental to the wound healing process, but it also impedes successful secondary fascial closure, which is a key factor in reducing mortality related to open abdomen therapy.[8, 12, 13] The size and location of an enteroatmospheric fistula can cause high-output proximal fistulae, leading to significant electrolyte and fluid loss and impairing the patient's nutrition, which can result in short bowel syndrome necessitating long-term parenteral nutrition and supplementation. [13] Enteroatmospheric fistula development therefore not only negatively affects morbidity and mortality, but has also been shown to be an independent predictor of significantly impaired quality of life. [14]

### Risk factors for enteroatmospheric fistula development

It is necessary to consider what risk factors may be associated with the development of enteroatmospheric fistulae. Technical factors during open abdomen therapy management, such as negative-pressure wound therapy, mesh interposition, and VPL use, as well as intestinal conditions that impede healing have been suggested as possible risk factors. [8, 11] Other risk factors include long open abdomen therapy duration and number of changing procedures, colonic resection, large oral fluid intake, low serum albumin, total parenteral nutrition and mesenteric ischaemia. [4, 11, 15, 16]

### The use of negative-pressure wound therapy and the occurrence of enteroatmospheric fistulae

The use of negative-pressure wound therapy and its duration during open abdomen therapy may influence the incidence of enteroatmospheric fistula. One study found that a long negative-pressure wound therapy duration was associated with a higher frequency of enteroatmospheric fistula. [17] Additionally, others suggested that negative-pressure wound therapy without VPL could decrease blood flow in the intestinal wall, and thus increase the risk of enteroatmospheric fistula. [18] However another experimental study revealed no macroscopic or microscopic changes to the intestinal tissue when a porous VPL foil was applied with negative-pressure wound therapy. [19] Enteroatmospheric fistulae were found to occur later, but in a more complex manner, if negative-pressure wound therapy was used in open abdomen therapy due to intraabdominal sepsis. [20] The enteroatmospheric fistula occurred in the negative-pressure wound therapy group later (median 18 days vs. 8 days without negative-pressure wound therapy, p=0.004). Enteroatmospheric fistula occurred after a median of 19 days for negative-pressure wound therapy, and after 17 days without negative-pressure wound therapy (p=0.59).

A recent Cochrane review concluded that it is uncertain whether negative-pressure wound therapy is superior to Bogota bag treatment in enteroatmospheric fistula [5]. This might, however, be largely explained by the fact that the Cochrane review only included two low-powered trials with a total of 75 patients. There are also good arguments that negative-pressure wound therapy is associated with reduced enteroatmospheric fistula incidence, likely due to the shorter duration of open abdomen therapy, particularly when used with Vacuum-Assisted Wound Closure and Mesh-Mediated Fascial Traction (VAWCM) techniques [6, 21]. Case studies have further revealed that negative-pressure wound therapy, when used in accordance with the VAWCM principle, displays lower enteroatmospheric fistula rates and significantly earlier and higher overall fascial closure rates [2, 22–26]. It is therefore accepted that a long duration of open abdomen therapy is to be avoided and that early secondary fascial closure is beneficial. [27] The question is not whether negative-pressure wound therapy should be used but how it should be used. Nearly all studies that showed beneficial effects of negative-pressure wound therapy also used concomitant VPL. [6]

### Mesh-mediated fascial traction and the use of a visceral protective layer

When utilising dynamic closure techniques, in particular mesh-mediated traction, it is imperative to remember that direct contact between mesh material and oedematous or granulating intestinal serosa without a VPL should be avoided given the increased risk of complications. [24, 25] Granulation, which is an early component of wound healing, is often intense and can lead to adhesions between the intestines and the mesh, as well as between the intestines themselves. If the delicate intestines are pressed against the mesh due to increased intra-abdominal pressure or the use of negative-pressure wound therapy, the mesh may migrate into the serosa. During the next dressing change, the removal of the mesh may cause lesions that result in the formation of enteroatmospheric fistula. Additionally, if negative-pressure wound therapy foams come into direct contact with the intestines, or if surgical towels or mesh materials are in contact with the intestines for an extended period of time, enteroatmospheric fistula may form due to the lack of inertness of these materials when in contact with the intestinal surface. It is essential to avoid any direct contact of the intestines to any non-inert material during open abdomen therapy to prevent enteroatmospheric fistula development. Therefore, we would recommend the use of a VPL as part of a standardised open abdomen therapy protocol.

### Practical aspects of the application of a visceral protective layer

This study, being the largest collective based on data from the EHS Open Abdomen Registry, shows that the use of a VPL is essential for preventing enteroatmospheric fistula during open abdomen therapy. When selecting a VPL for abdominal negative-pressure wound therapy, a range of suitable materials are available. For example, the ABThera™ Abdominal Dressing system from KCI Medical, San Antonio/TX USA, includes a specifically designed VPL with embedded foam. Additionally, individual products such as hydropolymers made of corn starch (PolyMem® WIC, Agentur Scherrer, Platznas/Switzerland) or special drainage films (Suprasorb® CNP drainage film, Lohmann & Rauscher, Neuwied/Germany) can be utilised. [19, 28] It is important to ensure that the entire intestines are covered when applying the VPL, as adhesions may develop to the lateral abdominal wall, potentially impeding or preventing a fascial closure. In our view, VPL should be embedded in a standardised treatment protocol for open abdomen therapy. This also has to include negative-pressure wound therapy and dynamic closure techniques because of their unique beneficial effects and the synergy with VPL in order to reduce the inherent risks of open abdomen therapy and utilise the strategy to its optimum benefit. The benefits of negative-pressure wound therapy (reduction of intestinal oedema, easy-access to the abdomen, creation of a contamination-protected wound compartment and granulation) are combined with avoidance of lateral fascial retraction. Ideally, adhesion to the abdominal wall and retraction of the fascial edges are thereby avoided and secondary fascial closure can be achieved as quickly as possible. [24, 29]

### Need for further evaluation of technical aspects of open abdomen therapy

To the best of our understanding, the investigation by Willms et al. is the only one to focus on the effect of VPL utilisation on the incidence of enteroatmospheric fistula in open abdomen therapy. [25] The EHS guideline only provides recommendation for VPL use at the level of expert guidance. [30, 31] Due to the heterogenous indications, comorbidities, and surgical management of open abdomen therapy strategies, it is difficult to compare them. To address these difficulties and to conduct well-designed analyses, registry data is the most efficient way to gather a large database. Apart from the EHS Open Abdomen registry, there is only one other registry (IROA) for open abdomen therapy. [2, 10]

**Supplementary Figures and Tables**

| **Patient characteristics** | | | | | | | | |
| --- | --- | --- | --- | --- | --- | --- | --- | --- |
|  | | Unmatched population | | | | Propensity score matched population | | |
|  | | All | Visceral protective layer | No visceral protective layer | p | Visceral protective layer | No visceral protective layer | p |
| N | | 1009 | 561 (55.6%) | 448 (44.4%) |  | 198 | 198 |  |
| Sex | m | 652 (65.9%) | 375 (67.1%) | 277 (64.3%) | .351 | 130 (66.3%) | 131 (66.2%) | .973 |
|  | f | 338 (34.1%) | 184 (32.9%) | 154 (35.7%) |  | 66 (33.7%) | 67 (33.8%) |  |
| Age [years] | | 59.8 ± 16.4 | 59.5 ± 16.5 | 60.2 ±16.4 | .544 | 59.2 ± 15.6 | 61.3 ± 16.3 | .206 |
| BMI [kg/m²] | | 27.4 ± 6.7 | 27.5 ± 6.9 | 27.3 ± 6.3 | .716 | 27.3 ± 6.3 | 26.6 ± 5.2 | .341 |
| Comorbidities | | 718 (71.2%) | 390 (69.5%) | 328 (73.2%) | .201 | 137 (69.9%) | 156 (78.8%) | .043 |
| Arterial hypertension | | 336 (33.3%) | 214 (38.1%) | 122 (27.2%) | .001 | 76 (38.8%) | 61 (30.8%) | .102 |
| Malignant disease | | 210 (20.8%) | 84 (15.0%) | 126 (28.1%) | .001 | 41 (20.9%) | 68 (34.3%) | .001 |
| Renal disease | | 142 (14.1%) | 66 (11.8%) | 76 (17.0%) | .024 | 26 (13.3%) | 34 (17.2%) | .279 |
| Haemodialysis | | 32 (3.2%) | 11 (2.0%) | 21 (4.7%) | .013 | 3 (1.5%) | 8 (4.0%) | .127 |
| Cardiac disease | | 282 (27.9%) | 141 (25.1%) | 141 (31.5%) | .030 | 55 (28.1%) | 74 (37.4%) | .051 |
| Pulmonary disease | | 146 (14.5%) | 98 (17.5%) | 48 (10.7%) | .001 | 21 (10.7%) | 23 (11.6%) | .777 |
| Diabetes | | 139 (13.8%) | 78 (13.9%) | 61 (13.6%) | .903 | 29 (14.8%) | 29 (14.6%) | .967 |
| Hepatic disease | | 39 (3.9%) | 16 (2.9%) | 23 (5.1%) | .062 | 3 (1.5%) | 9 (4.5%) | .078 |
| Other comorbidity | | 227 (22.5%) | 124 (22.1%) | 103 (23.1%) | .731 | 35 (17.9%) | 45 (22.7%) | .229 |
| Smoking  Packyears | | 139 (13.8%)  38.5 ± 23.3 | 79 (14.1%)  37.5 ± 18.8 | 60 (13.4%)  39.5 ± 26.8 | .750  .750 | 34 (17.3%)  44.4 ± 18.8 | 30 (15.2%)  46.4 ± 23.0 | .551  .827 |
| Risk factors  (immunosuppression and/or anticoagulants) | | 328 (32.5%) | 160 (28.5%) | 168 (37.5%) | .001 | 60 (30.6%) | 85 (42.9%) | .011 |
| Immunosuppressants | | 48 (4.8%) | 26 (4.6%) | 22 (4.9%) | .844 | 9 (4.6%) | 11 (5.6%) | .664 |
| Long-term corticosteroids | | 43 (4.3%) | 27 (4.8%) | 16 (3.6%) | .332 | 6 (3.1%) | 11 (5.6%) | .220 |
| Anti-coagulants | | 232 (23.0%) | 118 (21.0%) | 114 (25.4%) | .101 | 49 (25.0%) | 61 (30.8%) | .201 |
| Phenprocoumon | | 32 (3.2%) | 23 (4.1%) | 9 (2.0%) | .061 | 10 (5.1%) | 7 (3.5%) | .444 |
| Novel oral anticoagulants | | 19 (1.9%) | 4 (0.7%) | 15 (3.3%) | .003 | 0 (0.0%) | 9 (4.5%) | .002 |
| Heparin | | 50 (5.0%) | 28 (5.0%) | 22 (4.9%) | .952 | 9 (4.6%) | 12 (6.1%) | .420 |
| Platelet aggregation inhibitors | | 131 (13.0%) | 71 (12.7%) | 60 (13.4%) | .731 | 33 (16.8%) | 31 (15.7%) | .751 |

Table S1 - Patient characteristics, comorbidities, and risk factors associated with complications of open abdomen therapy.

| **Supplemental overview of surgical variables** | | | | | | | |
| --- | --- | --- | --- | --- | --- | --- | --- |
|  | Unmatched population | | | | Propensity score-matched population | | |
|  | All | Visceral protective layer | No visceral protective layer | p | Visceral protective layer | No visceral protective layer | p |
| N | 1009 (100.0%) | 561 (55.6%) | 448 (44.4%) |  | 198 | 198 |  |
| Previous surgery  Bowel resection with anastomosis  Bowel resection without anastomisis  Splenectomy  Pancreatic necrosectomy  Trauma exploration  Other | 237 (23.5%)  140 (13.9%)  21 (2.1%)  48 (4.8%)  112 (11.1%)  476 (47.2%) | 114 (20.3%)  92 (16.4%)  5 (0.9%)  21 (3.7%)  68 (12.1%)  274 (48.8%) | 123 (27.5%)  48 (10.7%)  16 (3.6%)  27 (6.0%)  44 (9.8%)  202 (45.1%) | .007  .008  .002  .086  .252  .244 | 53 (27.0%)  26 (13.3%)  3 (1.5%)  5 (2.6%)  12 (8.1%)  109 (55.6%) | 73 (36.9%)  21 (10.6%)  8 (4.0%)  9 (4.5%)  25 (12.6%)  82 (41.4%) | .044  .421  .131  .290  .028  .014 |
| Type of incision  Midline  Transverse  Combined  Laparoscopy | 712 (70.6%)  74 (7.3%)  22 (2.2%)  2 (0.2%) | 418 (74.5%)  35 (6.2%)  12 (2.1%)  1 (0.2%) | 294 (65.6%)  39 (8.7%)  10 (2.2%)  1 (0.2%) | .303 | 140 (71.4%)  20 (10.2%)  8 (4.1%)  0 (0.0%) | 148 (74.7%)  15 (7.6%)  3 (1.5%)  1 (0.5%) | .244 |
| Cause of peritonitis  Perforation  Anastomotic insuffiiciency  Pancreatitis  Other | 170 (50.4%)  87 (8.6%)  39 (11.6%)  41 (12.2%) | 113 (55.4%)  44 (21.6%)  15 (7.4%)  32 (15.7%) | 57 (42.9%)  43 (32.3%)  24 (18.0%)  9 (6.8%) | .001  .323  .033  .004 | 43 (51.2%)  30 (35.7%)  3 (3.6%)  8 (9.5%) | 24 (38.1%)  27 (42.9%)  8 (12.7%)  4 (6.3%) | .012  .642  .130  .229 |
| Mannheim Peritonitis Index | 15.7 ± 10.0 | 15.7 ± 10.2 | 15.7 ± 9.9 | .98 | 15.4 ± 8.6 | 15.2 ± 9.1 | .932 |
| Injury Severity Score | 37.1 ± 16.5 | 39.4 ± 15.8 | 31.1 ± 17.2 | .056 | 38.6 ± 11.1 | 32.6 ± 19.7 | .458 |
| APACHE-II Score | 18.8 ± 9.4 | 18.2 ± 9.1 | 19.4 ± 9.6 | .221 | 16.5 8.9 | 19.1 ± 9.5 | .139 |
| Sepsis at beginning of open abdomen therapy | 400 (39.6%) | 232 (41.5%) | 168 (37.5%) | .212 | 77 (38.9%) | 77 (38.9) | .943 |

Table S2 Additional variables that describe surgical and technical aspects leading to the requirement for open abdomen therapy.

| **2016 Björck Classification** | | | | | | | |
| --- | --- | --- | --- | --- | --- | --- | --- |
|  | Unmatched population | | | | Propensity score-matched population | | |
|  | All | Visceral protective layer | No visceral protective layer | p | Visceral protective layer | No visceral protective layer | p |
| Initial  IA  IB  IC  IIA  IIB  IIC  IIIA  IIIB | N=643  195 (30.3%)  121 (18.8%)  6 (0.9%)  84 (13.1%)  192 (28.9%)  11 (1.7%%)  3 (0.5%)  31 (4.8%) | N=414  140 (33.5%)  107 (25.8%)  5 (1.2%)  37 (8.9%)  111 (26.8%)  4 (1.0%)  0 (0.0%)  11 (2.6%) | N=234  51 (24.4%)  14 (6.1%)  1 (0.4%)  47 (20.5%)  81 (35.4%)  7 (3.1%)  3 (1.3%)  20 (8.7%) | <.001 | N=131  25 (19.1%)  30 (22.9%)  0 (0.0%)  19 (14.5%)  53 (40.5%)  0 (0.0%)  0 (0.0%)  4 (3.1%) | N=123  28 (22.7%)  7 (5.7%)  0 (0.0%)  29 (23.6%)  44 (35.8%)  3 (2.4%)  2 (1.6%)  10 (8.1%) | .001 |
| End of open abdomen therapy  IA  IB  IC  IIA  IIB  IIC  IIIA  IIIB  IV | N=504  203 (40.3%)  30 (6.0%)  0 (0.0%)  165 (32.7%)  44 (8.7%)  5 (1.0%)  38 (7.5%)  1 (0.0%)  18 (3.6%) | N=317  139 (43.8%)  19 (6.0%)  0 (0.0%)  113 (35.6%)  17 (3.0%)  1 (0.3%)  21 (6.7%)  0 (0.0%)  7 (2.2%) | N=187  64 (34.2%)  11 (5.9%)  0 (0.0%)  52 (27.8%)  27 (14.4%)  4 (2.1%)  17 (9.1%)  1 (0.5%)  11 (5.9%) | .001 | N=115  36 (31.3%)  8 (4.1%)  0 (0.0%)  53 (46.1%)  5 (4.3%)  0 (0.0%)  9 (7.8%)  0 (0.0%)  4 (3.5%) | N=115  36 (31.3%)  10 (8.7%)  0 (0.0%)  34 (29.6%)  17 (14.8%)  1 (0.9%)  11 (9.6%)  0 (0.0%)  6 (5.2%) | .078 |

Table S3 - 2016 Björck classification of the open abdomen. [3] Grade 1, without adherence between bowel and abdominal wall or fixity of the abdominal wall (lateralisation), subdivided as follows: 1A, clean; 1B, contaminated; and 1C, with enteric leak. An enteric leak controlled by closure, exteriorisation into a stoma, or a permanent enterocutaneous fistula is considered clean. Grade 2, developing fixation, subdivided as follows: 2A, clean; 2B, contaminated; and 2C, with enteric leak. Grade 3, frozen abdomen, subdivided as follows: 3A clean and 3B contaminated. Grade 4, an established enteroatmospheric fistula is defined as a permanent enteric leak into the open abdomen, associated with granulation tissue.

| **Supplemental outcome variables** | | | | | | | |
| --- | --- | --- | --- | --- | --- | --- | --- |
|  | Unmatched population | | | | Propensity score-matched population | | |
|  | All | Visceral protective layer | No visceral protective layer | p | Visceral protective layer | No visceral protective layer | p |
| N | 1009 | 561 | 448 |  | 198 | 198 |  |
| Ventilator hours | 245.0 ± 282.6 | 229.8 ± 252.9 | 266.0 ± 319.8 | .132 | 257.9 ± 294.9 | 252.3 ± 283.4 | .933 |
| Fascial closure  Definitive one-stage closure  Step-by-step closure | 561 (55.6%)  381 (37.8%)  180 (17.8%) | 369 (65.8%)  235 (41.9%)  134 (23.9%) | 192 (42.9%)  146 (32.6%)  46 (10.3%) | <.001 | 149 (76.0%)  90 (45.9%)  59 (30.1%) | 108 (54.5%)  75 (37.9%)  33 (16.7%) | .001 |
| Fascial separation [cm] at end of therapy | 4.6 ± 6.1 | 4.3 ± 5.9 | 5.3 ± 6.5 | .242 | 4.0 ± 4.8 | 5.2 ± 5.3 | .267 |
| Other complications  Clavien-Dindo  Total  <III  ≥III  IIIa  IIIb  IVa  IVb | 372 (36.9%)  110 (10.9%)  262 (26.0%)  45 (4.5%)  122 (12.1%)  33 (3.3%)  62 (6.1%) | 197 (35.1%)  75 (13.4%)  122 (21.7%)  21 (3.7%)  70 (12.5%)  16 (2.9%)  15 (2.7%) | 175 (34.2%)  35 (7.9%)  140 (31.3%)  24 (5.4%)  52 (11.6%)  17 (3.8%)  47 (10.5%) | .167  .008  .001  .221  .669  .404  .002 | 86 (43.4%)  28 (14.3%)  58 (29.3%)  4 (2.0%)  42 (21.4%)  6 (3.1%)  6 (3.1%) | 96 (48.5%)  17 (8.6%)  79 (39.9%)  17 (8.6%)  35 (17.7%)  12 (6.1%)  15 (7.6%) | .356  .081  .023  .002  .351  .154  .064 |

Table S4 - Additional outcome variables including Clavien-Dindo classification of complications.

**References**

References

1. Muysoms F, Campanelli G, Champault GG, DeBeaux AC, Dietz UA, Jeekel J, et al. EuraHS: The development of an international online platform for registration and outcome measurement of ventral abdominal wall hernia repair. Hernia. 2012;16:239–50. doi:10.1007/s10029-012-0912-7.

2. Willms A, Muysoms F, Güsgen C, Schwab R, Lock J, Schaaf S, et al. The Open Abdomen Route by EuraHS: Introduction of the data set and initial results of procedures and procedure-related complications. Hernia. 2017;21:279–89. doi:10.1007/s10029-017-1572-4.

3. Björck M, Kirkpatrick AW, Cheatham M, Kaplan M, Leppäniemi A, Waele JJ de. Amended Classification of the Open Abdomen. Scand J Surg. 2016;105:5–10. doi:10.1177/1457496916631853.

4. Cristaudo AT, Hitos K, Gunnarsson R, DeCosta A. Development and validation of a multivariable prediction model in open abdomen patients for entero-atmospheric fistula. ANZ J Surg. 2022;92:1079–84. doi:10.1111/ans.17512.

5. Cheng Y, Wang K, Gong J, Liu Z, Gong J, Zeng Z, Wang X. Negative pressure wound therapy for managing the open abdomen in non-trauma patients. Cochrane Database Syst Rev. 2022;5:CD013710. doi:10.1002/14651858.CD013710.pub2.

6. Atema JJ, Gans SL, Boermeester MA. Systematic review and meta-analysis of the open abdomen and temporary abdominal closure techniques in non-trauma patients. World J Surg. 2015;39:912–25. doi:10.1007/s00268-014-2883-6.

7. Tolonen M, Mentula P, Sallinen V, Rasilainen S, Bäcklund M, Leppäniemi A. Open abdomen with vacuum-assisted wound closure and mesh-mediated fascial traction in patients with complicated diffuse secondary peritonitis: A single-center 8-year experience. J Trauma Acute Care Surg. 2017;82:1100–5. doi:10.1097/TA.0000000000001452.

8. Websky MW von, Jedig A, Willms A, Jafari A, Matthaei H, Kalff JC, Manekeller S. Prognosefaktoren der offenen Abdominalbehandlung in der Viszeralchirurgie. Zentralbl Chir. 2017;142:259–66. doi:10.1055/s-0042-119303.

9. Tsuei BJ, Skinner JC, Bernard AC, Kearney PA, Boulanger BR. The open peritoneal cavity: Etiology correlates with the likelihood of fascial closure. Am Surg. 2004;70:652–6.

10. Sibilla MG, Cremonini C, Portinari M, Carcoforo P, Tartaglia D, Cicuttin E, et al. Patients with an Open Abdomen in Asian, American and European Continents: A Comparative Analysis from the International Register of Open Abdomen (IROA). World J Surg. 2023;47:142–51. doi:10.1007/s00268-022-06733-4.

11. Coccolini F, Ceresoli M, Kluger Y, Kirkpatrick A, Montori G, Salvetti F, et al. Open abdomen and entero-atmospheric fistulae: An interim analysis from the International Register of Open Abdomen (IROA). Injury. 2019;50:160–6. doi:10.1016/j.injury.2018.09.040.

12. Scott BG, Welsh FJ, Pham HQ, Carrick MM, Liscum KR, Granchi TS, et al. Early aggressive closure of the open abdomen. J Trauma. 2006;60:17–22. doi:10.1097/01.ta.0000200861.96568.bb.

13. Wainstein DE, Calvi RJ, Rezzonico F, Deforel ML, Perrone N, Sisco P. Management of enteroatmospheric fistula: A ten-year experience following fifteen years of learning. Surgery 2023. doi:10.1016/j.surg.2022.12.001.

14. Theodorou A, Jedig A, Manekeller S, Willms A, Pantelis D, Matthaei H, et al. Long Term Outcome After Open Abdomen Treatment: Function and Quality of Life. Front Surg. 2021;8:590245. doi:10.3389/fsurg.2021.590245.

15. Bradley MJ, Dubose JJ, Scalea TM, Holcomb JB, Shrestha B, Okoye O, et al. Independent predictors of enteric fistula and abdominal sepsis after damage control laparotomy: Results from the prospective AAST Open Abdomen registry. JAMA Surg. 2013;148:947–54. doi:10.1001/jamasurg.2013.2514.

16. Giudicelli G, Rossetti A, Scarpa C, Buchs NC, Hompes R, Guy RJ, et al. Prognostic Factors for Enteroatmospheric Fistula in Open Abdomen Treated with Negative Pressure Wound Therapy: A Multicentre Experience. J Gastrointest Surg. 2017;21:1328–34. doi:10.1007/s11605-017-3453-7.

17. Mintziras I, Miligkos M, Bartsch DK. High risk of fistula formation in vacuum-assisted closure therapy in patients with open abdomen due to secondary peritonitis-a retrospective analysis. Langenbecks Arch Surg. 2016;401:619–25. doi:10.1007/s00423-016-1443-y.

18. Lindstedt S, Malmsjö M, Hansson J, Hlebowicz J, Ingemansson R. Microvascular blood flow changes in the small intestinal wall during conventional negative pressure wound therapy and negative pressure wound therapy using a protective disc over the intestines in laparostomy. Ann Surg. 2012;255:171–5. doi:10.1097/SLA.0b013e31823c9ffa.

19. Auer T, Wiederstein-Grasser I, Sauseng S, Delcev P, Preisegger KH. The Effect of Negative Pressure in the Abdominal Cavity With Suprasorb CNP on Abdominal Organs-An Experimental Study. Front Surg. 2020;7:584926. doi:10.3389/fsurg.2020.584926.

20. Kalaiselvan R, Slade DAJ, Soop M, Burnett H, Lees NP, Anderson ID, et al. Impact of negative pressure wound therapy on enteroatmospheric fistulation in the septic open abdomen. Colorectal Dis 2022. doi:10.1111/codi.16318.

21. Coccolini F, Biffl W, Catena F, Ceresoli M, Chiara O, Cimbanassi S, et al. The open abdomen, indications, management and definitive closure. World J Emerg Surg. 2015;10:32. doi:10.1186/s13017-015-0026-5.

22. Rasilainen S, Mentula P, Salminen P, Koivukangas V, Hyöty M, Mäntymäki L-M, et al. Superior primary fascial closure rate and lower mortality after open abdomen using negative pressure wound therapy with continuous fascial traction. J Trauma Acute Care Surg. 2020;89:1136–42. doi:10.1097/TA.0000000000002889.

23. Willms A, Güsgen C, Schaaf S, Bieler D, Websky M von, Schwab R. Management of the open abdomen using vacuum-assisted wound closure and mesh-mediated fascial traction. Langenbecks Arch Surg. 2015;400:91–9. doi:10.1007/s00423-014-1240-4.

24. Willms AG, Schwab R, Websky MW von, Berrevoet F, Tartaglia D, Sörelius K, et al. Factors influencing the fascial closure rate after open abdomen treatment: Results from the European Hernia Society (EuraHS) Registry : Surgical technique matters. Hernia. 2022;26:61–73. doi:10.1007/s10029-020-02336-x.

25. Willms AG, Schaaf S, Zimmermann N, Schwab R, Güsgen C, Vilz TO, et al. The Significance of Visceral Protection in Preventing Enteroatmospheric Fistulae During Open Abdomen Treatment in Patients With Secondary Peritonitis: A Propensity Score-matched Case-control Analysis. Ann Surg. 2021;273:1182–8. doi:10.1097/SLA.0000000000003440.

26. Becker HP, Willms A, Schwab R. Small bowel fistulas and the open abdomen. Scand J Surg. 2007;96:263–71. doi:10.1177/145749690709600402.

27. Gasser E, Rezaie D, Gius J, Lorenz A, Gehwolf P, Perathoner A, et al. Lessons Learned in 11 Years of Experience With Open Abdomen Treatment With Negative-Pressure Therapy for Various Abdominal Emergencies. Front Surg. 2021;8:632929. doi:10.3389/fsurg.2021.632929.

28. Auer T, Sauseng S, Delcev P, Kohek P. Effect of Negative Pressure Therapy on Open Abdomen Treatments. Prospective Randomized Study With Two Commercial Negative Pressure Systems. Front Surg. 2020;7:596056. doi:10.3389/fsurg.2020.596056.

29. Mahoney EJ, Bugaev N, Appelbaum R, Goldenberg-Sandau A, Baltazar GA, Posluszny J, et al. Management of the open abdomen: A systematic review with meta-analysis and practice management guideline from the Eastern Association for the Surgery of Trauma. J Trauma Acute Care Surg. 2022;93:e110-e118. doi:10.1097/TA.0000000000003683.

30. López-Cano M, García-Alamino JM, Antoniou SA, Bennet D, Dietz UA, Ferreira F, et al. EHS clinical guidelines on the management of the abdominal wall in the context of the open or burst abdomen. Hernia 2018. doi:10.1007/s10029-018-1818-9.

31. Bruhin A, Ferreira F, Chariker M, Smith J, Runkel N. Systematic review and evidence based recommendations for the use of negative pressure wound therapy in the open abdomen. Int J Surg. 2014;12:1105–14. doi:10.1016/j.ijsu.2014.08.396.
